# Enhanced Triboelectric Charge Stability by Air‐Stable Radicals

**DOI:** 10.1002/advs.202304459

**Published:** 2023-09-07

**Authors:** Sooik Im, Ethan Frey, Daniel J. Lacks, Jan Genzer, Michael D. Dickey

**Affiliations:** ^1^ Department of Chemical and Biomolecular Engineering North Carolina State University Raleigh NC 27695‐7905 USA; ^2^ Department of Chemical and Biomolecular Engineering Case Western Reserve University Cleveland OH 44106 USA

**Keywords:** air‐stable radicals, charge retention, kelvin probe force microscopy (KPFM), self‐assembled monolayer (SAM), triboelectric charge

## Abstract

This paper demonstrates that air‐stable radicals enhance the stability of triboelectric charge on surfaces. While charge on surfaces is often undesirable (e.g., static discharge), improved charge retention can benefit specific applications such as air filtration. Here, it is shown that self‐assembled monolayers (SAMs) containing air‐stable radicals, 2,2,6,6‐tetramethylpiperidin‐1‐yl)oxidanyl (TEMPO), hold the charge longer than those without TEMPO. Charging and retention are monitored by Kelvin Probe Force Microscopy (KPFM) as a function of time. Without the radicals on the surface, charge retention increases with the water contact angle (hydrophobicity), consistent with the understanding that surface water molecules can accelerate charge dissipation. Yet, the most prolonged charge retention is observed in surfaces treated with TEMPO, which are more hydrophilic than untreated control surfaces. The charge retention decreases with reducing radical density by etching the TEMPO‐silane with tetrabutylammonium fluoride (TBAF) or scavenging the radicals with ascorbic acid. These results suggest a pathway toward increasing the lifetime of triboelectric charges, which may enhance air filtration, improve tribocharging for patterning charges on surfaces, or boost triboelectric energy harvesting.

## Graphical abstract

This paper reports a strategy for enhancing triboelectric charge stability by depositing air‐stable radicals on a surface. By utilizing AFM probes to precisely induce triboelectric charge on self‐assembled monolayers, it is demonstrated that air‐stable radicals help triboelectric charge sustain ≈3x longer than without the radicals. This concept opens up the possibility to extend the lifetime of air filtration systems, including masks.

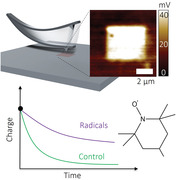

## 1 Introduction

Triboelectric charging can occur when two materials are in contact and pulled apart. The phenomenon can be observed in daily life. Examples include the electric shock felt when grabbing a doorknob during the winter or lightning caused by electrostatic discharge from the atmosphere. While first reported more than two thousand years ago, the fundamental understanding of the triboelectric effect is limited due to the stochastic nature of a charge on insulators, which makes it hard to quantify.^[^ ^1^, ^2^ ^]^ The mechanism of tribocharging on insulators is still under debate; it is unclear whether it is caused by the transfer of electrons,^[^ ^3^, ^4^, ^5^, ^6^ ^]^ ions,^[^ ^7^, ^8^, ^9^ ^]^ or materials.^[^ ^10^, ^11^, ^12^, ^13^ ^]^

Another unclear issue of triboelectric charge is how charges dissipate over time.^[^ ^14^ ^]^ Previous studies have found that charges can dissipate due to diffusion of charge to an uncharged surface,^[^ ^15^ ^]^ drift by surface conductance,^[^ ^16^, ^17^ ^]^ UV exposure,^[^ ^18^, ^19^ ^]^ breakdown by air, or via water molecules.^[^ ^20^, ^21^ ^]^ Water molecules adsorbed onto the surface accelerate charge dissipation.^[^ ^15^ ^]^ Since charge breakdown is a potential risk of damaging electronic devices,^[^ ^22^ ^]^ recent studies have mainly focused on removing charge,^[^ ^11^, ^23^ ^]^ or resisting charging.^[^ ^8^, ^24^ ^]^

Instead of removing or resisting charge, herein, we work on enhancing the stability. Enhanced tribocharging could be used in air filtration to trap micro‐size particles by electrostatic interaction,^[^ ^25^, ^26^, ^27^ ^]^ to assemble charged particles.^[^ ^28^ ^]^ or to improve triboelectric energy harvesting.^[^ ^29^ ^]^ We hypothesized that intentionally depositing radicals on the surface could increase the lifetime of tribocharges.^[^ ^30^, ^31^ ^]^ The hypothesis is based on prior work that shows radical scavengers can prevent tribocharging.^[^ ^11^ ^]^ Yet, to date, the intentional addition of radicals has not been explored to enhance tribocharging.

**Figure 1:**
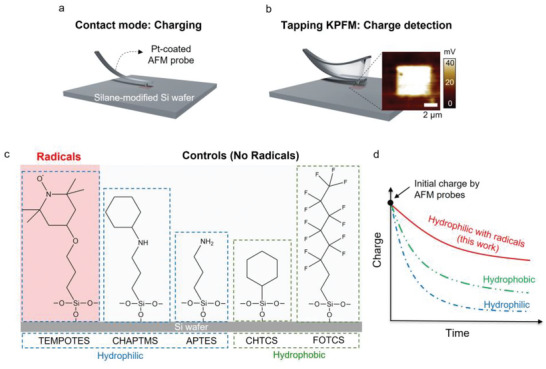
Schematic diagram of quantification of triboelectric charge on a surface‐modified Si wafer using a) contact mode atomic force microscopy (AFM) and b) non‐contact tapping mode KPFM. c) Si wafers were modified with different silanes, including 4‐(triethoxysilylpropoxy)−2,2,6,6‐tetramethylpiperidine N‐oxide (TEMPOTES), (N‐cyclohexylaminopropyl)trimethoxysilane (CHAPTMS), (3‐aminopropyl)triethoxysilane (APTES), trichloro(cyclohexyl)silane (CHTCS), and trichloro(1H, 1H, 2H, 2H‐perfluoro‐octyl)silane (FOTCS). d) Comparison of charge decay with different surface chemistries with or without radicals.

This study investigates charge retention on various silane‐modified Si wafers, including (2,2,6,6‐tetramethylpiperidin‐1‐yl)oxyl (TEMPO) containing air‐stable nitroxide radicals.^[^ ^32^ ^]^ (**Figure** **1**) With the air‐stable radicals, tribocharges on the surface were retained for more than 12 h, which is longer than untreated samples. Interestingly, increased hydrophilicity – such as that achieved by depositing TEMPO—typically decreases charge stability. Yet, we show herein that the TEMPO‐containing surfaces deviate significantly from the typical relationship between charge retention and surface hydrophobicity (Figure 1d). We also tuned radical density on the surface using two strategies: 1) tetrabutylammonium fluoride (TBAF) to etch TEMPO‐silane (TEMPOTES) from the surface^[^ ^33^ ^]^ and 2) ascorbic acid to remove the radicals^[^ ^34^ ^]^ and investigate the effect of radicals on charge retention.

## 2 Results and Discussion

### 2.1 Charge Retention

Charging and retention were characterized by Kelvin probe force microscopy (KPFM), a type of atomic force microscopy (AFM) used to quantify surface potential on the dielectric surface.^[^ ^35^ ^]^ Figure 1a,b show a schematic diagram of contact mode charging the surface and detection with KPFM. In contact mode, Pt‐coated cantilevers were brought into contact with surface‐modified Si wafers to tribocharge a 4 × 4 µm^2^ area. After charging the surface, we measured the charge decay using non‐contact KPFM over an 8 × 8 µm^2^ area to distinguish between charged and uncharged areas. The area was scanned with KPFM continuously to track charge dissipation over time.

**Figure 2:**
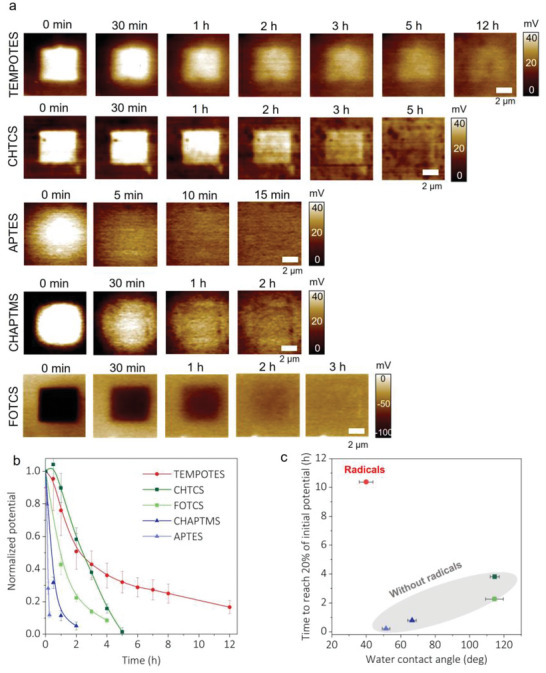
Charge dissipation over time with different silanes on Si wafers. The relative humidity is maintained at 30–40%. a) KPFM images to monitor the dissipation of triboelectric charge on TEMPOTES, CHTCS, APTES, CHAPTMS, and FOTCS. b) Comparison of charge dissipation by normalized potential. The lines are meant to guide the eye. c) The relationship between time to reach 20% of initial potential and water contact angles of each silane‐treated surface. Generally, the lower the contact angle, the less time it takes to dissipate the charge, except for TEMPOTES, which contains radicals.

**Figure** **2**a shows KPFM potential images with different silane treatments to track charge retention over time. In all cases, a rectangular pattern was formed on the areas contacted by AFM probes. The silanes were tribocharged by the AFM probes without changing the surface topography (Figure S1, Supporting Information). To estimate the charge on each surface, we assume the relative permittivity of silane molecules is ≈2.1. This value was taken from C8–C14 alkanethiol monolayers,^[^ ^36^ ^]^ assuming that silane molecules have similar relative permittivity. The thickness of SiO_2_ is ≈5 nm after oxygen plasma treatment for 1 min (Figure S4, Supporting Information), and the thickness of silanes is assumed to be 0.5 nm. By a parallel plate model (detail in the experiment section), TEMPOTES (20.66 ± 4.3 nC cm^−2^), CHTCS (19.83 ± 1.8 nC cm^−2^), APTES (14.21 ± 2.1 nC cm^−2^), CHAPTMS (39.64 ± 5.7 nC cm^−2^) were positively charged, whereas FOTCS (−48.79 ± 5.7 nC cm^−2^) was negatively charged. The charge sign on each surface follows the triboelectric series, a “heuristic table” describing the general trend of charging signs when two materials are brought in contact.

To highlight the role of radicals, we tested a silane with a TEMPO‐like molecular structure that lacked radicals (CHAPTMS). Among the silanes, the surface with TEMPOTES showed the best charge retention of ≈12 h. Charge on CHAPTMS decayed within ≈1 h (similar to other silanes that lack radicals), an order of magnitude faster than that of TEMPOTES. It suggests that stable radicals on TEMPOTES play an essential role in charge retention.

Figure 2b summarizes the charge retention on different silanes over time by normalizing the surface potential. Charges on hydrophobic silanes, including FOTCS and CHTCS, show moderate charge retention for ≈3–4 h to reach 10% of their initial potential. In contrast, CHAPTMS reached the same level within ≈1 h. This difference in behavior may be explained by decreased adsorption of water molecules on hydrophobic surfaces,^[^ ^37^ ^]^ which minimizes the chance of charge dissipation by surface water.^[^ ^15^ ^]^

Figure 2c plots charge retention time versus the water contact angle of silane‐treated Si wafers. We find that the more hydrophobic the surface, the longer the charge retention, as expected.^[^ ^38^ ^]^ There was one exception: TEMPOTES. TEMPOTES is hydrophilic and should have dissipated charge rapidly. Instead, it held the charge for over ≈12 h. It further indicates that the radicals seem to help stabilize the tribocharge.

As mentioned in the introduction, there are multiple mechanisms by which charge can dissipate from surfaces. Our studies do not conclusively provide insight into the mechanism of charge dissipation, but we offer a few considerations for future research. The AFM images in Figure 2 show that the charge stays localized to the initial square‐shaped charge region rather than spread laterally. Thus, the charge appears to either dissipate through or at the surface of the monolayer rather than via lateral motion.

To model the charge dissipation, we started with the conservation of charge to model charge dissipation with different silanes. We assumed the charge acts as a parallel capacitor (Q = C_0_U). Other KPFM studies used the same assumption, and the modeling results matched prior experimental results.^[^ ^15^, ^39^ ^]^ Here, we assumed two ways to dissipate charge: 1) lateral diffusion by charge gradients across the surface and 2) "reaction". Reaction is a generic term that captures the disappearance of charge via mechanisms such as air breakdown or surface conduction. Equation 1 shows these terms as a reaction‐diffusion system:^[^ ^40^, ^41^ ^]^

(1) $$\;\frac{\partial U}{\partial t}=\;D\left(\frac{\partial^{2}U}{\partial x^{2}}\right)-kU$$

where *U* is surface potential (V), *D* is diffusion coefficient (µm^2^ s^−1^), and *k* is reaction constant (s^‐1^). Here, we assume a first‐order reaction. Equation 1 was solved numerically using Finite Difference Approximations.^[^ ^15^ ^]^ *D* and *k* were determined by fitting the calculated curves with experimental data (Figures S2–S3, Supporting Information). From this analysis, charge dissipation is mainly caused by “reaction” (e.g., surface conduction or air breakdown), not by diffusion (Figure S4, Supporting Information). Diffusion coefficients of different surfaces are within the range from ≈10^−7^ to ≈5 × 10^−6^ µm^2^ s^−1^. As expected, TEMPOTES shows the lowest reaction constant, meaning that tribocharge on TEMPOTES possessed the best charge retention among these silane treatments.

Given the negligible lateral transport of charge across the surface, the charge must dissipate through or on the surface. The AFM experiments we used require a grounded silicon wafer. Yet, the 5 nm oxide layer (Figure S4, Supporting Information) on the wafer is a barrier to charge transport between the surface. Thus, if the charge dissipates through the surface, it will require tunneling. Alternatively, the charge could dissipate at the surface via breakdown by interaction with molecules from the air, such as water.^[^ ^15^, ^20^, ^21^ ^]^

### 2.2 Tuning Radical Density

**Figure 3:**
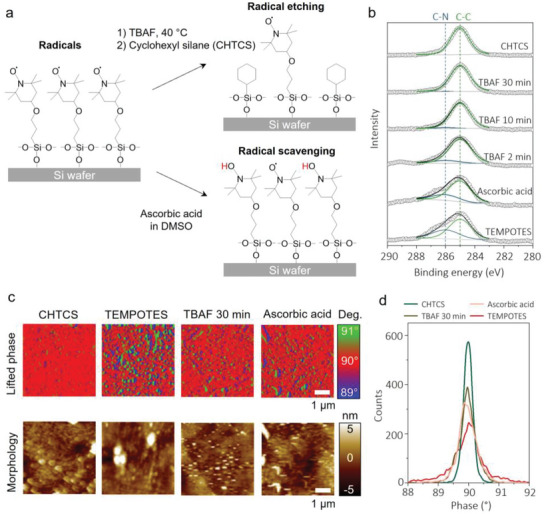
Tuning the density of air‐stable radicals on the surface. Schematic diagram of surface treatment with a) TBAF or ascorbic acid. The TBAF etches the TEMPOTES, and the ascorbic acid “scavenges” the radicals. b) High‐resolution C1s survey of TBAF‐ or ascorbic acid‐treated surface. c) Lifted phase and morphology images using MFM to characterize the presence of radicals on the surface and d) phase distribution of each sample.

**Figure** **3**a shows two different approaches to tuning the density of air‐stable radicals on the surface. TBAF was used to etch TEMPOTES from Si wafers. By tuning the exposure time with TBAF, the amount of etched TEMPOTES can be controlled.^[^ ^33^ ^]^ CHTCS backfilled the exposed portions of the surface. Another approach to decrease radicals on TEMPOTES is to treat TEMPO with ascorbic acid, a known radical scavenger.^[^ ^34^ ^]^ TEMPOTES was exposed to ascorbic acid for 6 h before measurement to help quench any radicals on TEMPOTES.

Figure 3b shows an XPS spectrum of high‐resolution C1s with different surface treatments to determine how the two approaches modified the surface. TEMPOTES (bottom) shows a prominent C─C peak at 285 eV and a shoulder C─N peak at ≈286 eV.^[^ ^42^ ^]^ After ascorbic acid treatment, these two peaks remained, indicating that ascorbic acid does not affect the chemical structure of TEMPOTES. Whereas 2 min of TBAF treatment followed by backfilling of CHTCS reduced the intensity of the C‐N peak associated with TEMPO. Increasing the exposure time to 30 min made the C─N peak mostly disappear, resulting in C1s results nearly identical to CHTCS. A quantitative analysis of the change in the intensities of the C─N peak is shown in Table S1 (Supporting Information). The high‐resolution N1s also showed a new peak at 397.7 eV after treatment with ascorbic acid, indicating that N‐O·partially converted to N‐OH, as expected^[^ ^43^ ^]^ (Figure S5, Supporting Information).

Magnetic force microscopy (MFM) mapped the radicals on the surface. The magnetic interaction between magnetized probe tips and radicals on the surface shifts the frequency of oscillating probes. These are shown in phase scans, which are not affected by surface topography. Although, in principle, electron paramagnetic resonance (EPR) or electron spin resonance (ESR) can measure radicals, prior studies have shown that such techniques produce a poor signal‐to‐noise ratio due to the small amount of material in a monolayer coating.^[^ ^44^, ^45^ ^]^ Previous studies have relied on MFM to characterize radicals, in which a magnetized AFM tip interacts with radicals as it moves across the surface.^[^ ^11^, ^46^, ^47^ ^]^ We use this technique to detect the presence of radicals. Figure 3c shows the lifted phase images and topography of CHTCS, TEMPOTES, and TBAF‐ or ascorbic acid‐treated silanes. The phase deviation from 90° implies magnetic interaction between the tip and surface, a measure of the presence of radicals. In the case of TEMPOTES, blue (below 90°) and green (above 90°) dots were more distinct than those from any other images, confirming that the radicals are present on the surface. Also, blue and red dots are next to each other; such radicals have dipolar properties, which were similarly reported in studies on radical‐containing polymers^[^ ^46^ ^]^ and magnetic particles.^[^ ^47^, ^48^ ^]^ These MFM results only provide the presence of radicals before the contact between a probe and the surface. However, we reason that radicals remain active because 1) the morphology of the TEMPOTES‐treated surface does not change due to the contact (Figure S1, Supporting Information), and 2) TEMPO radicals are chemically stable at ambient conditions. The radicals decompose only in acidic environments with elevated temperatures (> 80 °C).^[^ ^49^ ^]^

**Figure 4:**
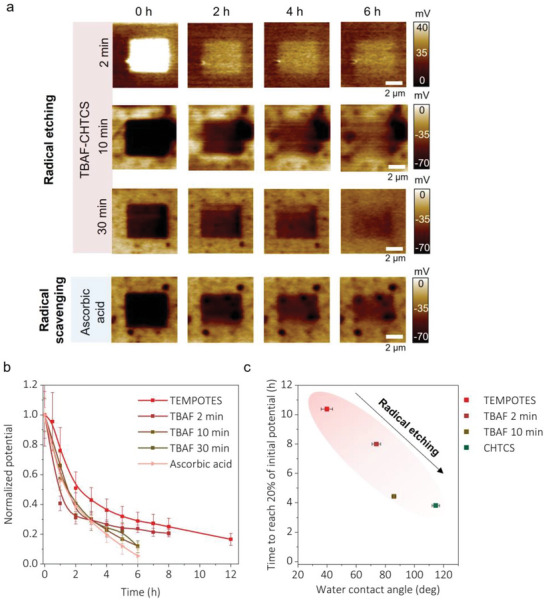
Charge retention with different radical densities on the surface. a) KPFM images of charge retention over time with TBAF/CHTCS‐ and ascorbic acid‐treated TEMPOTES. b) Normalized charge retention over time with different treatments on TEMPOTES. The lines are meant to guide the eye. c) The relationship between time to reach 20% of initial potential and water contact angles of TEMPOTES, CHTCS, and TBAF/CHTCS‐treated TEMPOTES.

**Figure** **4** shows charge retention on TBAF‐ and ascorbic acid‐treated TEMPOTES. As shown in Figure 4a, the surfaces were tribocharged positively when treated with TBAF for less than 10 min. The sign of the charge reversed to negative when the exposure time was increased to more than 10 min. It could be explained by the presence of fluorine from TBAF attaching to the Si wafer, which was confirmed by an F1s peak in an XPS survey (Figure S6, Supporting Information). After the ascorbic acid treatment, the surface was negatively charged, suggesting that the conversion N‐O· to N‐OH could also cause a reverse in the charge sign. Also, the sign reversal might indicate that tribocharging cannot be solely explained by electron transfer. N‐OH is typically an electron‐donating group, which means the surface should have been positively charged if the electron transfer were the only mechanism to explain the origin of tribocharge. Instead, the negative charge could imply ion transfer from the substrate.

Figure 4b summarizes charge retention on TBAF and ascorbic acid‐treated surfaces over time by normalizing the surface potentials. Compared to charge retention on TEMPOTES, the charge decays faster, indicating that removing radicals reduces the temporal stability of the triboelectric charge.

Figure 4c relates charge retention time with the water contact angle of TBAF‐treated TEMPOTES and CHTCS. With radicals, the more hydrophobic the surface, the shorter the charge retention, which shows the opposite trend from Figure 2c. Radical density significantly affects charge retention. In summary, air‐stable radicals could stabilize triboelectric charge. This finding agrees with a previous study in which mechano‐radicals stabilized triboelectric charge.^[^ ^11^ ^]^ A follow‐up study using molecular simulations suggests that radicals can stabilize charge through the interactions of radicals with the molecular orbital of positive/negative charges on the surface, lowering the energy levels of the charges and making them more stable.^[^ ^30^ ^]^

## 3 Conclusions

We show that air‐stable radicals on a surface enhance triboelectric charge retention by using KPFM. Without the radicals (TEMPOTES), charge retention increases with hydrophobicity (increasing water contact angle) because water molecules can accelerate charge dissipation. However, hydrophilic TEMPOTES stayed charged for more than 12 h, which deviates significantly from the expectation of charge retention based on hydrophilicity alone. This result suggests that radicals play a crucial role in stabilizing charge. The radical density on the surface was tuned by TBAF treatment to etch TEMPOTES or ascorbic acid treatment to remove radicals selectively. XPS and MFM confirmed that the radical density decreased after these treatments. Charge retention measurements on these modified surfaces showed charges dissipate faster with decreasing radical density. This study suggests a new yet simple way to enhance tribocharge stability using air‐stable radicals. The work has potential implications for improved filtration (e.g., face masks and air filters in cars or homes) or enhanced triboelectric energy harvesting by simple silanization of surfaces. It could also offer routes to pattern stable charges on surfaces for various applications, such as the self‐assembly of charged particles or proteins.

## 4 Experimental Section

### Materials

Isopropanol (IPA), dimethyl sulfoxide (DMSO), tetrabutylammonium fluoride (TBAF) in tetrahydrofuran (THF), and L‐ascorbic acid were purchased from Sigma‐Aldrich. For silane treatments on Si wafers (University Wafers, orientation <100>), 4‐(triethoxysilylpropoxy)−2,2,6,6‐tetramethylpiperidine N‐oxide (TEMPOTES) (Gelest), trichloro(cyclohexyl)silane (CHTCS) (Gelest), (3‐aminopropyl) triethoxysilane (APTES) (Sigma‐Aldrich), Trichloro(1H, 1H, 2H, 2H‐perfluoro‐octyl)silane (FOTCS) (Sigma‐Aldrich), and (N‐cyclohexylaminopropyl)trimethoxysilane (CHAPTMS) (Gelest) were used.

### Sample Preparation: Silane Treatments on Si Wafers

Silicon wafers were cleaned with IPA and oxygen‐plasma treated for 1 min. (Oxygen plasma, Diener) 5 µL of silanes was dropped and put in the petri dish under the vacuum for 30 min. Silane‐treated Si wafers were washed with IPA multiple times to remove unreacted silanes on the surface and then put in the vacuum oven at 60 °C overnight. Before the measurement, the samples were placed in Si wafer carriers for at least 1 day at room temperature.

### Sample Preparation: TBAF Treatment

TEMPO‐treated Si wafers were dipped into 0.1 м TBAF in THF at 40 °C for different exposure times and then washed several times with IPA and toluene to remove residual TBAF thoroughly. Then CHTCS was back‐filled on TBAF‐treated samples. After washing with IPA, the samples were kept with the same procedure.

### Sample Preparation: Ascorbic Acid Treatment

TEMPO‐treated Si wafers were dipped into 0.1 m ascorbic acid in DMSO for several hours. The samples were washed and handled with the same procedure as TBAF treatment.

### Charge Generation and Detection

For charge generation and detection, AFM (Asylum MFP‐3D, Oxford Instruments) was used with Ti/Pt‐coated Si‐based probes (AC240TM‐R3, Oxford Instruments) with a nominal resonance frequency of 70 kHz and the spring constant of 2 N m^−1^. Relative humidity in the AFM chamber was maintained at 30–40% by CaCl_2_ salts. The triboelectric charge was generated in the contact mode by adjusting the set point to control the contact force. In the contact mode, no external bias was applied to create a charge only by the physical contact. Generated surface potential increased with increasing the contact force (Figure S7, Supporting Information). We chose the contact force of ≈1.7 µN in the rest of the experiments because it generated moderate surface potential (≈40 mV) without affecting the surface topography. KPFM detected the surface potentials at 50 nm above the topography scan (non‐contact tapping mode). To obtain accurate surface potential in KPFM, the lifted height of the AFM tip from the substrate should be higher than the surface roughness to remove any surface interactions between the tip and substrate that could affect the frequency of the probes. In all our samples, the root‐mean‐square surface roughness (S_q_) was less than 5 nm, an order of magnitude lower than the lifted height (50 nm). In this mode, a DC voltage is applied to the probe to nullify oscillating electrical forces generated by the potential difference between the probe and the substrate. The sign of “surface potential” depends on the convention of the AFM: in our measurements, the reported signs (+/‐) of surface potential refer to the sign of surface charge.^[^ ^50^ ^]^ Prior studies have converted potential (V) to charge per area (σ) by assuming the surface is like a capacitor (Q = CV).^[^ ^51^, ^52^ ^]^ We assume SiO_2_ and silanes form series capacitance.

(2) $$\sigma \;=\;\;\frac{\Delta V\varepsilon_{0}\varepsilon_{r}}{d}=\frac{\Delta V\varepsilon_{0}}{\frac{d_{1}}{\varepsilon_{1}}+\frac{d_{2}}{\varepsilon_{2}}}\;$$

where Δ*V* is a potential difference between charged and uncharged area. *ε*_0_ is vacuum permittivity, and *ε*_r_ is the relative permittivity of the material. *d* is the thickness of the dielectric layer. Subscripts 1 and 2 denote SiO_2_ and silane molecules, respectively.

The conversion only works when the charged area (16 µm^2^) is much larger than the thickness of the insulators (≈5 nm), which is valid in our case. If the charged area is similar to the thickness of insulators, or a surface has both positive and negative charge locally, a numerical simulation is required to determine Green's function of probes, insulators, and ground system to accurately quantify charge from surface potential.^[^ ^53^ ^]^ The probes were worn down after multiple contacts (Figure S8, Supporting Information) and were replaced with fresh ones regularly when the charging sign reversed.^[^ ^54^ ^]^ After obtaining 8 × 8 µm^2^‐sized KPFM images, surface potentials of the contact area were quantified by setting the surface potential of a non‐contact area as a baseline. Then the surface potential of the contact area was averaged. All measurements were repeated three times to confirm reproducibility.

### Radical Detection

In MFM, Co‐Cr magnetized AFM probes (NSC18/Co‐Cr, MikroMasch) were used to detect radicals on the surface with a lifting height of 40 nm. The drive amplitude for the lifted phase image was 50 mV. Since the lifting height and the drive amplitude could affect phase shift, in all cases, these parameters were fixed to compare the phase shifts of each sample in the same way.

### Surface Characterization

Variable angle spectroscopic ellipsometry (VASE, J.A. Woollam Co.) was employed to measure SiO_2_ thickness on Si wafers after oxygen plasma treatment. The measurements were carried out at two angles of incident light (67° and 72°, relative to the normal) with wavelengths between 400 and 1000 nm. X‐ray photoelectron spectroscopy (XPS) was carried out on a SPECS system with an Mg Kα source. Energy calibration was carried out using C1s with a binding energy of 285 eV. Field Emission Scanning Electron Microscope (FE‐SEM, FEI Verios 460L) was used to see the morphology of fresh and used AFM probes. The contact angle was measured by a goniometer (FTA 1000B Frame, First Ten Angstroms).

## Conflict of Interest

The authors declare no conflict of interest.

## Supporting information

Supporting Information

## Data Availability

The data that support the findings of this study are available from the corresponding author upon reasonable request.
