# Supplementary material for: Enhanced Triboelectric Charge Stability by Air‐Stable Radicals
Source: Adv Sci (Weinh). 2023 Sep 7;10(31):2304459. doi: 10.1002/advs.202304459 (PMC10625048; doi:10.1002/advs.202304459)
Supplement: Supplementary file 1 — Supporting Information [file ADVS-10-2304459-s001.pdf]

ADVANCED SCIENCE
Open Access

Supporting Information

for *Adv. Sci.*, DOI 10.1002/advs.202304459

Enhanced Triboelectric Charge Stability by Air-Stable Radicals

Sooik Im, Ethan Frey, Daniel J. Lacks, Jan Genzer and Michael D. Dickey**

Supporting Information

Enhanced triboelectric charge stability by air-stable radicals

Sooik Im[1], Ethan Frey[1], Daniel J. Lacks[2], Jan Genzer[1,*], Michael D. Dickey[1,*]

[1] Department of Chemical and Biomolecular Engineering, North Carolina State University

Raleigh, NC, USA

[2] Department of Chemical Engineering, Case Western Reserve University

Cleveland, OH, USA

Corresponding authors

mddickey@ncsu.edu

jgenzer@ncsu.edu

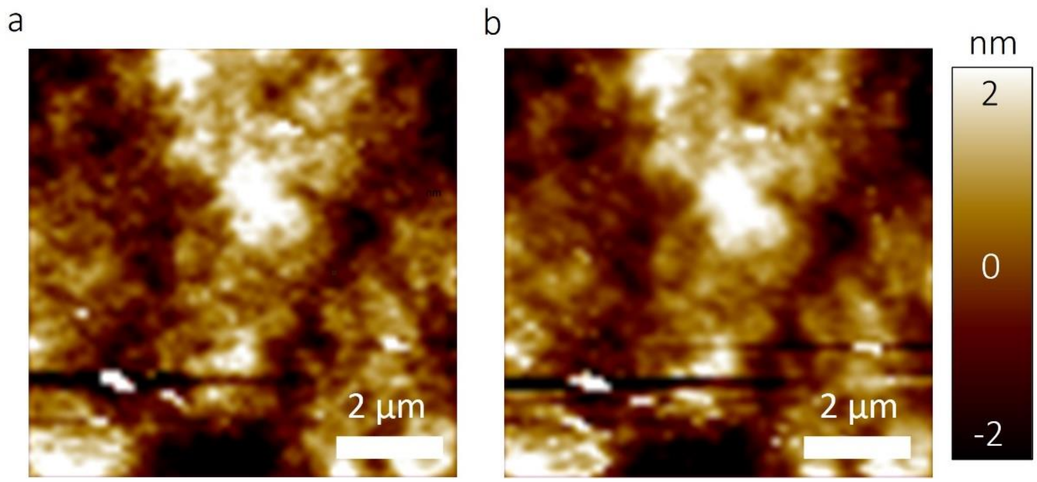

Figure S1. Surface topography (a) before and (b) after the contact with the AFM probe.

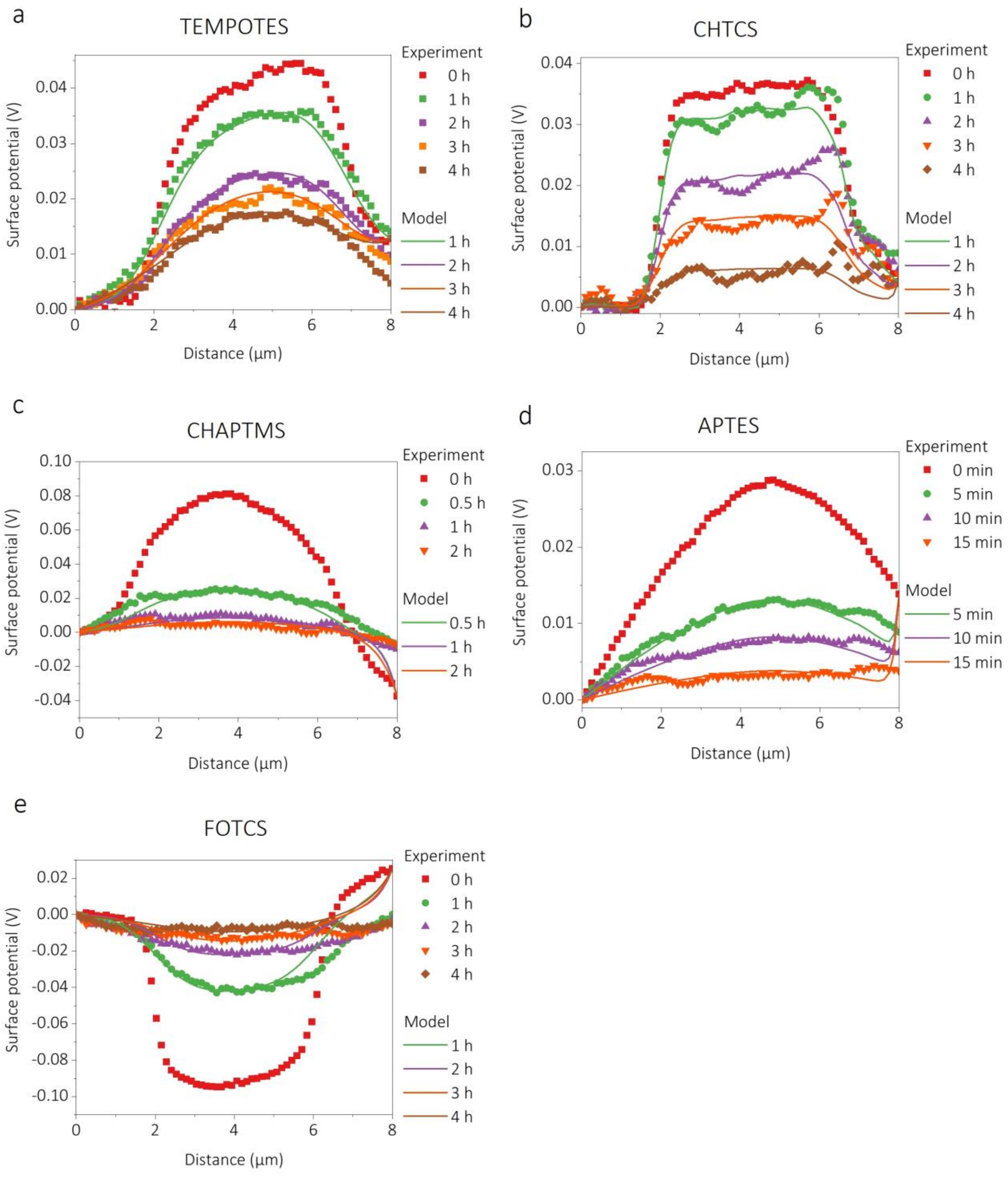

Figure S2. Surface potential profiles on the surface of (a) TEMPOTES, (b) CHTCS, (c) CHAPTMS, (d) APTES, and (e) FOTCS. The dotted lines are experimental data, and the solid lines are calculated using equation (1).

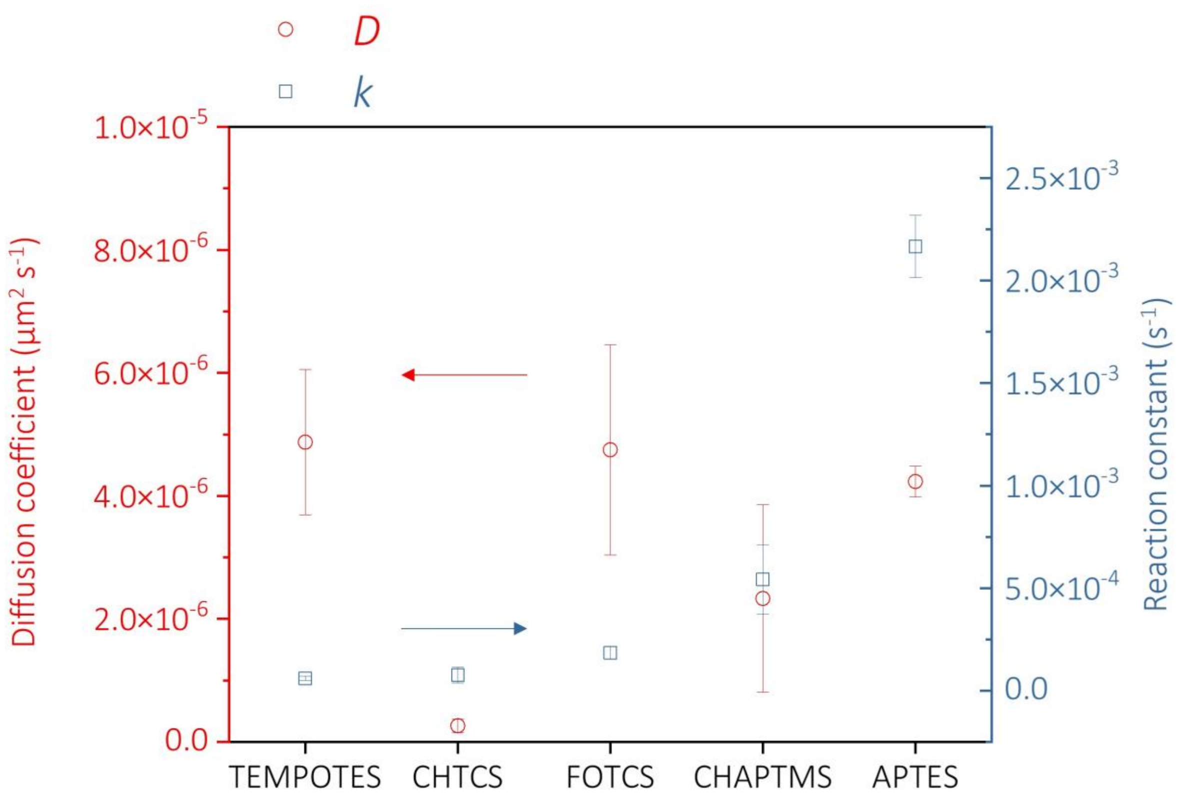

Figure S3. The estimated diffusion coefficients and reaction constants from the curves of surface potential profiles in Figure S2.

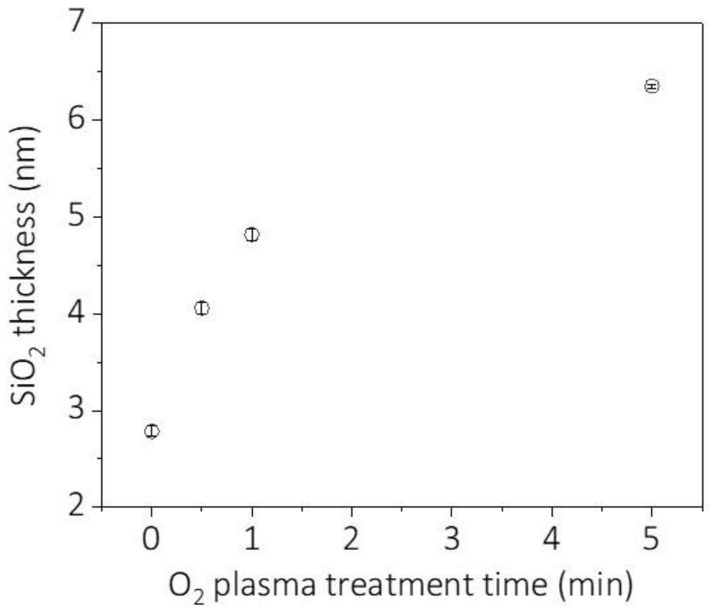

Figure S4. SiO_2 thickness with different O_2 plasma treatment times. The thickness was measured by variable angle spectroscopic ellipsometry (VASE). All experiments for silane treatments were done on Si wafers after O_2 plasma treatment for 1 min.

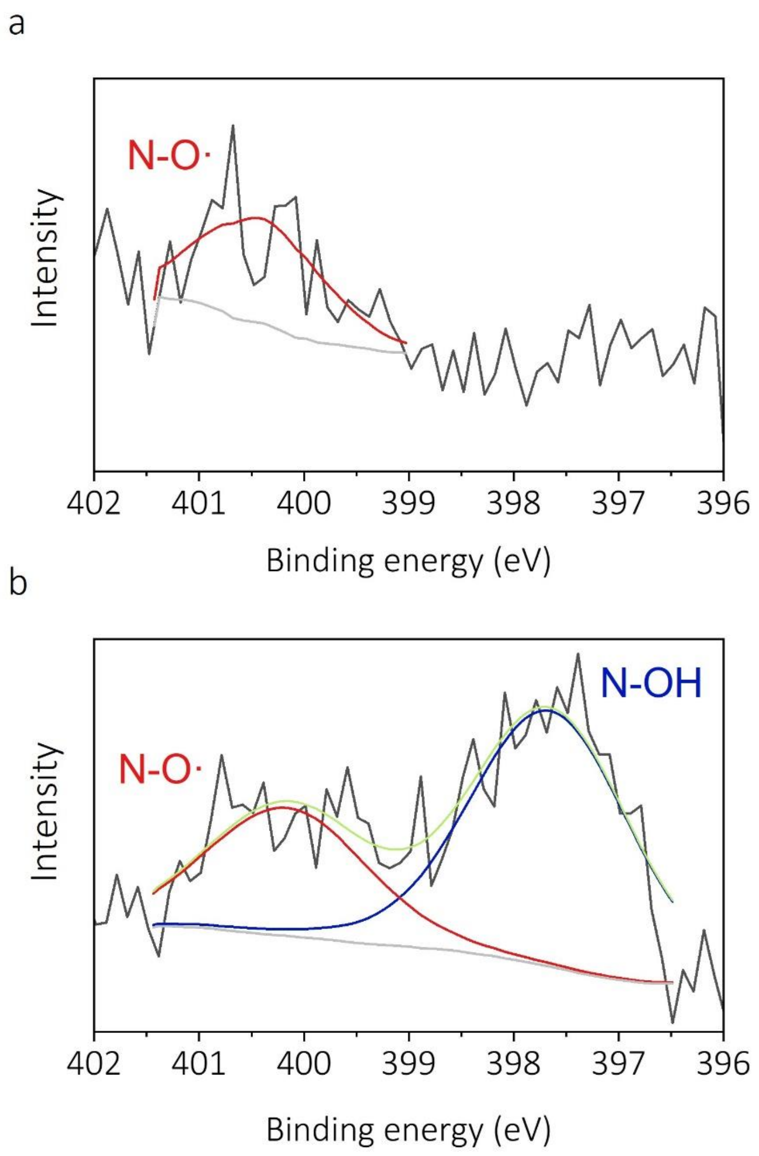

Figure S5. High-resolution XPS N1s spectra of (a) before and (b) after ascorbic acid treatment on TEMPOTES.

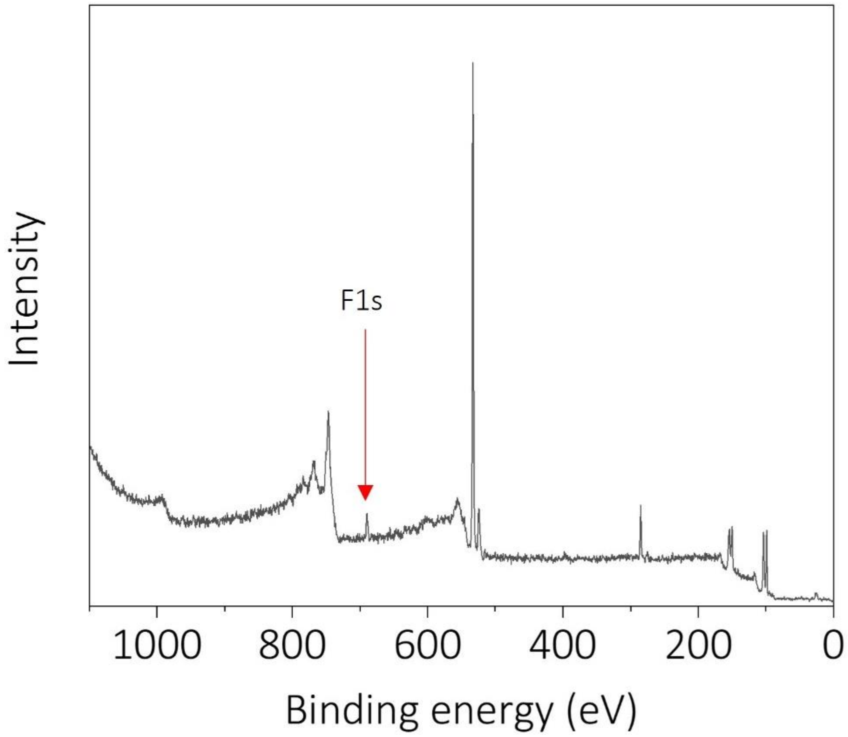

Figure S6. XPS survey of TBAF-CHTCS 10 min, showing F1s peak.

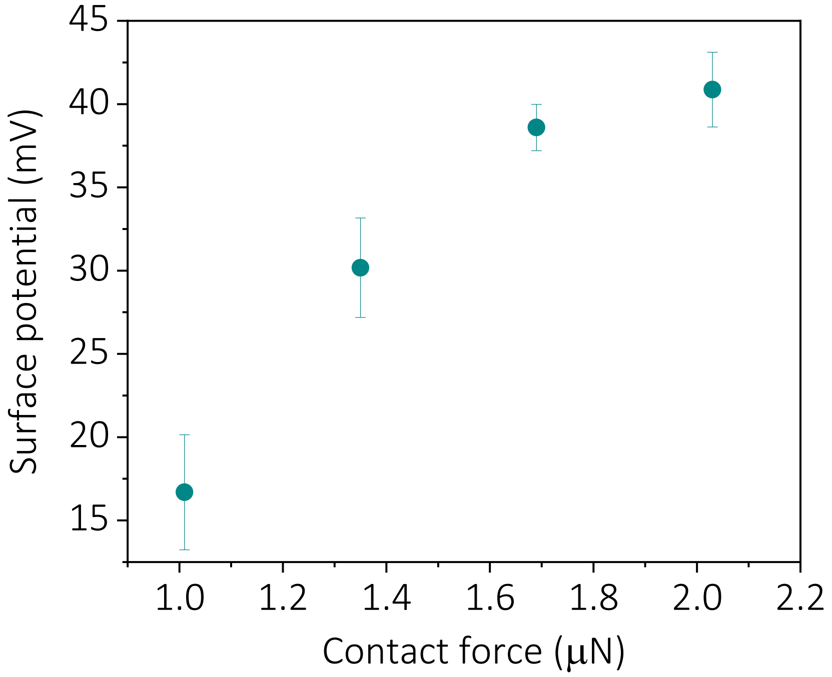

Figure S7. Surface potentials of TEMPOTES surface with different contact forces.

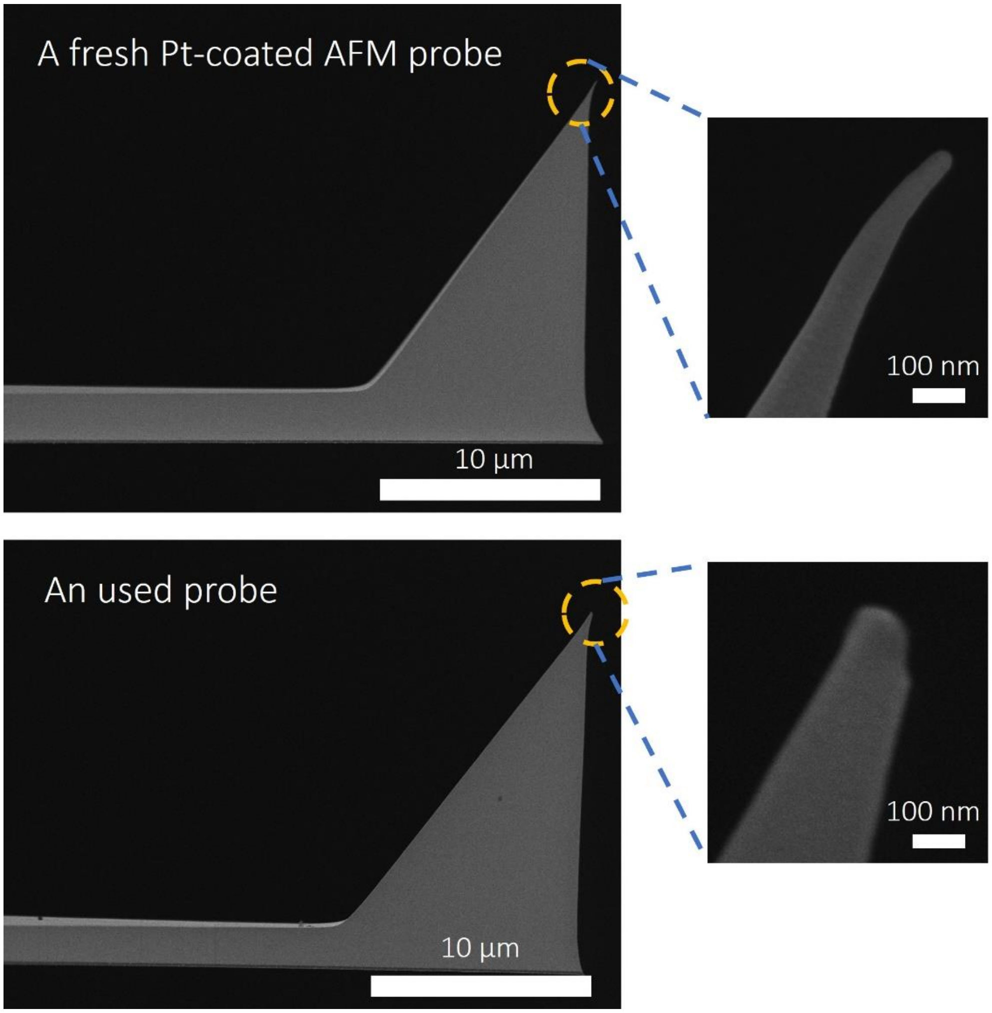

Figure S8. SEM images of (a) a fresh and (b) a used AFM probe. The probe was used for three different contact modes and ~30 h of non-contact tapping mode.

	**C-C [%]**	**C-N [%]**
TEMPOS	65	35
Ascorbic acid	75.16	24.84
TBAF 2 min	87.44	12.56
TBAF 10 min	96.14	3.86
TBAF 30 min	98.86	1.14
CHTCS	100	0

Table S1. Intensities of C-C and C-N components from high-resolution C1s XPS survey in Figure 3b.
